# Yeast cell detection using fuzzy automatic contrast enhancement (FACE) and you only look once (YOLO)

**DOI:** 10.1038/s41598-023-43452-9

**Published:** 2023-09-27

**Authors:** Zheng-Jie Huang, Brijesh Patel, Wei-Hao Lu, Tz-Yu Yang, Wei-Cheng Tung, Vytautas Bučinskas, Modris Greitans, Yu-Wei Wu, Po Ting Lin

**Affiliations:** 1 Department of Mechanical Engineering, National Taiwan University of Science and Technology, Taipei, 10607 Taiwan; 2 Vilnius Gediminas Technical University, 10223 Vilnius, Lithuania; 3 Institute of Electronics and Computer Science, Riga, 1006 Latvia; 4 Graduate Institute of Biomedical Informatics, College of Medical Science and Technology, Taipei Medical University, Taipei, 11031 Taiwan; 5 Clinical Big Data Research Center, Taipei Medical University Hospital, Taipei, 11031 Taiwan; 6 TMU Research Center for Digestive Medicine, Taipei Medical University, Taipei, 11031 Taiwan; 7 Intelligent Manufacturing Innovation Center, National Taiwan University of Science and Technology, Taipei, 10607 Taiwan

**Keywords:** Computational biology and bioinformatics, Microbiology, Engineering

## Abstract

In contemporary biomedical research, the accurate automatic detection of cells within intricate microscopic imagery stands as a cornerstone for scientific advancement. Leveraging state-of-the-art deep learning techniques, this study introduces a novel amalgamation of Fuzzy Automatic Contrast Enhancement (FACE) and the You Only Look Once (YOLO) framework to address this critical challenge of automatic cell detection. Yeast cells, representing a vital component of the fungi family, hold profound significance in elucidating the intricacies of eukaryotic cells and human biology. The proposed methodology introduces a paradigm shift in cell detection by optimizing image contrast through optimal fuzzy clustering within the FACE approach. This advancement mitigates the shortcomings of conventional contrast enhancement techniques, minimizing artifacts and suboptimal outcomes. Further enhancing contrast, a universal contrast enhancement variable is ingeniously introduced, enriching image clarity with automatic precision. Experimental validation encompasses a diverse range of yeast cell images subjected to rigorous quantitative assessment via Root-Mean-Square Contrast and Root-Mean-Square Deviation (RMSD). Comparative analyses against conventional enhancement methods showcase the superior performance of the FACE-enhanced images. Notably, the integration of the innovative You Only Look Once (YOLOv5) facilitates automatic cell detection within a finely partitioned grid system. This leads to the development of two models—one operating on pristine raw images, the other harnessing the enriched landscape of FACE-enhanced imagery. Strikingly, the FACE enhancement achieves exceptional accuracy in automatic yeast cell detection by YOLOv5 across both raw and enhanced images. Comprehensive performance evaluations encompassing tenfold accuracy assessments and confidence scoring substantiate the robustness of the FACE-YOLO model. Notably, the integration of FACE-enhanced images serves as a catalyst, significantly elevating the performance of YOLOv5 detection. Complementing these efforts, OpenCV lends computational acumen to delineate precise yeast cell contours and coordinates, augmenting the precision of cell detection.

## Introduction

In the realm of medical and biological research, the utilization of microscopic imagery capturing cells, tissues, and entire organisms has become a prevailing practice^1^. The rapid development of single-cell sequencing technologies makes it possible to explore cell heterogeneity of genome, epigenome, transcriptome, and cell interaction/communication in the context of a specific environment in a tissue. However, the accurate detection of individual cells within complex microscopic imagery presents significant challenges. Due to various technical noises such as dropout rate, accurately measuring the expression level in a single cell is pretty challenging. Human analysis can no longer sustain the high throughput image data^2^. In the era of artificial intelligence and machine learning, these cutting-edge approaches are now widely applied in biological image processing, such as cell type identification, cell count, cell structure analysis, and cell reconstruction^3^. On the other hand, the effectiveness of image classification algorithms and the quality of the extracted image characteristics limit the accuracy and robustness of most of the methods now in use.

The yeast cell, with its precise developmental requirements and genetic tractability, serves as a valuable model organism in the field of biology. Optical investigations of yeast cell structure play a crucial role across various microbiological research areas, encompassing cell cycle modelling and ageing dynamics studies^4–6^. The current landscape of image processing and segmentation techniques employed for yeast images predominantly relies on traditional methodologies^7^, including approaches such as thresholding, edge detection, contour fitting, and watershed algorithms. However, the segmentations^8^ produced by these techniques often necessitate frequent human interventions, as demonstrated in multiple tests. Challenges inherent in yeast cell picture segmentation include issues such as cell crowding, irregular shapes, transparent inclusions, anomalies in visible structures, budding events, and inconsistent imaging focus. Complicating matters further, yeast cell images obtained from diverse imaging devices, such as microscopes, exhibit low contrast^9^ due to various factors, including the nature of the specimen, image-capturing device calibration settings, and suboptimal ambient lighting conditions.

In recent years, the field of automatic yeast cell detection has been revolutionized by the emergence of deep learning techniques, particularly convolutional neural networks (CNNs)^10^. These modern approaches leverage large amounts of labelled data to train highly complex models capable of directly learning intricate patterns and features from images. Automatic yeast cell detection within images characterized by low contrast presents a substantial challenge in biological image analysis, where intricate details often become obscured, hampering accurate detection and subsequent analysis. Contrast enhancement is one of the methods of enhancing image quality and highlighting the intricacies of hidden data in the low-contrast regions of an image. Different intensity adjustments were applied to boost the visual contrast^11^. While enhancing the contrast depends on the selection of parameters of function coefficients that users have artificially determined. Most transformation techniques change the original intensity of the image to a new value, resulting in a distinct pattern. No single transformation function is ideal for improving contrast in each image^12^.

The task of automatic yeast cell detection presents inherent challenges within image analysis, often exacerbated by factors such as low contrast and intricate backgrounds. To overcome these challenges, this study proposes the utilization of image enhancement techniques emerges as a pivotal strategy to enhance yeast cell visibility and accentuate their distinctive attributes. Notably, the integration of Fuzzy Automatic Contrast Enhancement (FACE)^13^ stands out as an effective solution, optimizing contrast dynamics while preserving the inherent natural colour distribution. Moreover, the incorporation of the You Only Look Once (YOLO)^14^ framework, a sophisticated deep learning-based object detection methodology, serves to elevate the precision and efficiency of yeast cell identification. This combination of image enhancement and YOLO enables the precise identification and localization of yeast cells within complex biological images, contributing to the advancement of research in microbiology and biology by facilitating more accurate and insightful analyses of yeast cells in their complex environmental contexts. However, some limitations of the study include challenges tied to labelling, manpower, and time constraints during dataset preparation.

## Related work

### Image enhancement

Image enhancement plays a significant role in augmenting image quality by emphasizing crucial features, mitigating secondary ones, enriching information, and refining details are undeniable. This process can be broadly categorized into two groups^15^: spatial domain processing, which operates on individual pixels and then combines neighbouring ones; and frequency domain processing, which processes the entire image collectively. Recent advancements have introduced efficient algorithms for image enhancement, with many traditional techniques being supplanted by neural network-based models^16^. This study explores the utilization of various image enhancement models, such as Fuzzy Automatic Contrast Enhancement (FACE), Retinex Algorithm, and Histogram Equalization (HE), to enhance the image quality of cell images.

Histogram equalization, a contrast adjustment technique using image histograms, aids in enhancing global contrast in images^17,18^. Despite its effectiveness in addressing images with extreme brightness levels, it may not precisely adjust localized areas, resulting in overexposed or underexposed images with amplified noise^19^. The Retinex algorithm^20^, founded on retinex theory^21^, captures the interaction of illumination and reflection to determine object color^22,23^. Single Scale Retinex (SSR) and Multiple Scale Retinex (MSR) methods exhibit different enhancement outcomes, with MSR integrating SSR outputs using varying weights to address dynamic range limitations^24^. To overcome colour distortion issues, Multi-Scale Retinex with Color Restoration (MSRCR) emerges as a solution, surpassing MSR in imaging quality and enhancement. The Fuzzy Automatic Contrast Enhancement (FACE)^13^ technique employs Fuzzy C-Means (FCM)^25^ clustering for pixel classification, leveraging a universal contrast enhancement variable (UCEV) to automatically maximize image entropy. This approach ensures enhanced contrast without introducing artifacts or noise, benefiting from both accuracy and smoothness in color distribution.

This paper presents the FACE as a contrast enhancement technique for enhancing the microscopic images of yeast cells, changing the original image pixels to a more uncongested pixel distribution. The fuzziness will maintain the smoothness of colour distribution to prevent artifacts in yeast cell images. The maximization approach using a universal contrast enhancement variable was presented to have better contrast enhancement automatically without manually fed parameters. Root-Mean-Square Deviation (RMSD) and Root-Mean-Square Contrast (RMSC) are used to evaluate the contrast of images and the difference between before and after enhancement^26,27^.

### Cell detection

Biological research has substantially adopted Machine Learning (ML) and Deep Learning (DL), notably in creating automated computer vision tools for recognizing and classifying microscopic images, with a focus on species-level identification^28,29^. Various computer vision techniques have emerged in this context for cell recognition and detection. In cell detection images, challenges arise due to the visual similarity, close proximity, and overlapping nature of cells in the 2D representation of 3D structures^30^. Uneven illumination, low contrast, low resolution, and diverse foreground/background intensities further complicate the task. Object detection, especially in cell detection, revolves around precise segmentation for local and global differentiation^31^. Given the multitude of cells in these images, precise detection is essential, particularly in overlap scenarios.

Li et al.^32^ conducted a comprehensive review of methods for microorganism image analysis, encompassing preparation, feature extraction, classification, and evaluation. Rea et al.^33^ introduced a GPU-based software utilizing the simplex method for efficient yeast cell detection in time-lapse microscopy, offering real-time analysis capabilities. An advanced RIC-Unet neural network was developed for accurate nuclei segmentation in histology images, integrating residual blocks, multi-scale, and channel attention. Van Valen et al.^34^ employed the DeepCell software with CNNs for cell segmentation in diverse microscopy images, sharing training insights. Hilsenbeck et al.34 introduced fastER, an efficient cell segmentation tool using SVM to estimate cell likelihood from candidate regions with texture and shape features, adopting a divide and conquer approach for optimal region selection.

Li et al.^32^ extensively reviewed methods for analyzing microscope images of microorganisms, where he focused on techniques for classifying microorganisms using different image processing steps like preparation, feature extraction, classification, and evaluation. Rea et al.^33^develops a GPU-based software using the simplex method for the efficient detection of single yeast cells in time-lapse microscopy images. This approach offers significant speedup compared to CPU processing, enabling real-time analysis of microfluidics chip data. An advanced RIC-Unet neural network introduces accurate nuclei segmentation in histology images, incorporating techniques like residual blocks, multi-scale, and channel attention^34^. Van Valen et al. in^34,35^ use DeepCell software with CNNs for cell segmentation across diverse microscopy images, sharing training insights. Hilsenbeck et al.^34,36^ introduce fastER, an efficient cell segmentation tool using SVM to estimate cell likelihood from candidate regions with texture and shape features, using divide and conquer for optimal region selection.

Worldwide, researchers have been attracted to the fundamental and crucial object detection task in computer vision^37^. Deep learning, particularly deep convolutional neural networks (CNNs), can automatically classify input datasets, such as images. CNNs directly learn from raw pixel data and class labels through end-to-end learning. There are primarily three families of detectors for object detection using deep learning models. These models are Region-CNN(R-CNN)^38,39^, the Single Shot Detector (SSD)^40^ and the Yolo series^14^. However, it’s worth noting that despite the remarkable capabilities of deep learning models like R-CNN, SSD, and YOLO in object detection, they often encounter challenges when dealing with low-light images. The reduced visibility in such conditions can hinder the efficiency and accuracy of object detection processes using these models. So the effect of contrast enhancement on YOLOv5 object detection was studied in this research, and in conclusion, the object detection capabilities of yeast cell image patterns are compared.

## Materials and methods

### Dataset

To curate the dataset, we captured raw images using a commercially available 8-megapixel camera featuring a 1/1.8-inch sensor size and the SONY imx334 sensor (Pixel size: 2.0 × 2.0 μm). The camera was paired with a 14X-90X optical zoom achromatic field objective, seamlessly integrated into a custom-designed positioning system. To initiate the experimentation phase, a few drops of a solution containing yeast cells (Saccharomyces cerevisiae) were delicately placed onto a petri dish strategically positioned beneath the camera’s objective. This meticulous setup enabled the acquisition of high-resolution images, effectively capturing a diverse spectrum of yeast cells spanning various growth stages. After image capture, a meticulous post-processing regimen ensued, involving resizing these images to a standardized dimension of 500 pixels × 500 pixels. This resizing operation facilitated the creation of images depicting a reduced quantity of cells, thereby rendering them optimally suited for subsequent analytical procedures.

The resultant dataset encompassed a comprehensive compilation of 1000 raw microscopic images of yeast cells, all meticulously formatted to the dimensions of 500 pixels × 500 pixels. This dataset served as the foundational substrate for the FACE image enhancement model. In the initial phases, the FACE methodology was applied to enhance the contrast of these 1000 raw microscopic images, culminating in creation of an enriched and enhanced image dataset. Notably, this dual dataset, consisting of original raw images and their corresponding FACE-enhanced counterparts, formed the bedrock for the subsequent development and validation of the YOLOv5 cell detection model.

### Fuzzy automation contrast enhancement (FACE)

**Figure 1:**
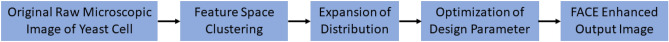
Schematic diagram of FACE.

Fuzzy Automatic Contrast Enhancement (FACE)^13^ is a unique method based on fuzzy clustering and a fuzzy expansion of pixel dispersion whose schematic diagram is shown in Fig. 1.

Initially, FACE utilizes a Fuzzy C-Means (FCM) clustering technique to segment an image while categorizing CIELAB colour space pixels with similar colours into tiny image clusters with appropriate attributes. Each group’s RGB colour space pixels are dispersed away from the respective cluster’s centre to enhance the image’s contrast, which maintains the consistency of pixel colours within each cluster. To automatically improve picture contrast, a Universal Contrast Enhancement Variable (UCEV) is created and adjusted to maximize the entropy of the image. A less cluttered distribution of the image pixels guaranteed more excellent picture contrast. The suggested entropy-maximization approach can enhance image quality without manually setting control parameters.

### Fuzzy C-Means clustering

Assuming there are $n$ image pixels $x_{i}$ for $i = 1...n$*,* and arranged into $k$ groups whose center is denoted by $j = 1...k$*.* The inclusion of the $i{\text{th}}$ pixel in $j{\text{th}}$ cluster denotes $u_{ij}$. K-means as an exact clustering method^41,42^, the value of $u_{ij}$ is either 0 or 1, where 0 stands for inclusion and 1 stand for non-inclusion. The Fuzzy C-Means (FCM) algorithm allows for imperfect pixel inclusiveness in each cluster. As it states, that $u_{ij}$ will be a real number with an interval of [0, 1] and always satisfy the Eq. (1).

1 $$\sum\limits_{j = 1}^{k} {u_{ij} } = 1$$

The definition of total in-group variance is shown in the Eq. (2).

2 $$J = \sum\limits_{j = 1}^{k} {\sum\limits_{j = 1}^{k} {\left( {u_{ij}^{m} \left\| {x_{i} - c_{j} } \right\|^{2} } \right)} }$$

Different researchers^43^ suggest the strength of inclusion parameters varies from 1 to 5, so for this study, the inclusion parameter is considered as $m = 3$. For optimal image segmentation, pixels $J$ must be minimized due to smaller in-group variations leading to larger ones^44^. The following formulation of a Lagrangian equation is as follows:

3 $$L = \sum\limits_{j = 1}^{k} {\sum\limits_{i = 1}^{n} {\left( {u_{ij}^{m} \left\| {x_{i} - c_{j} } \right\|^{2} + \lambda_{i} u_{ij} } \right)} } - \sum\limits_{i = 1}^{n} {\lambda_{i} }$$

where $\lambda_{i}$ is a Lagrangian multiplier, which was evaluated by the L minimization problem. The two equalities in the below-given Eqs. (4), and (5) can be satisfied to get the optimal fuzzy inclusiveness of each image pixel.

4 $$\frac{\partial L}{{\partial u_{ij} }} = mu_{ij}^{m - 1} \left\| {x_{i} - c{}_{j}} \right\|^{2} + \lambda_{i} = 0$$

5 $$\frac{\partial L}{{\partial c_{j} }} = \sum\limits_{i = 1}^{n} {(2u_{ij}^{m} (x_{i} - c_{j} )) = 0}$$

A parameterized method based on the Eqs. (1), (4), and (5) are demonstrated below, to evaluate the ideal parameters:

6 $$c_{j} = \frac{{\sum\nolimits_{i = 1}^{n} {u_{ij}^{m} x_{i} } }}{{\sum\nolimits_{i = 1}^{n} {u_{ij}^{m} } }}$$

7 $$u_{ij} = \frac{1}{{\sum\limits_{l = 1}^{k} {\left[ {\frac{{\left\| {x_{i} - c_{j} } \right\|}}{{\left\| {x_{i} - c_{l} } \right\|}}} \right]^{{\frac{2}{m - 1}}} } }}$$

Equation (6) states the center of the $j{\text{th}}$ cluster based on given inclusiveness, whereas the Eq. (7) states the inclusiveness parameter $u_{ij}$ based on the given cluster center points. The value of L converges until the parameterized process continues. In our approach, the parameterized technique begins with arbitrary assumptions given by $u_{ij}$, and the applied approach can produce consistent results across trials. The FCM generally produces the best identification findings in the CIELAB color space. The Commission International d’Eclairage established the CIELAB color space, where L stands for lightness and AB represents the color component dimensions.

### Entropy maximization concerning the universal contrast enhancement variable (UCEV)

The Universal Contrast Enhancement Variable (UCEV) is a pivotal concept within the framework of contrast enhancement, serving as a dynamic control parameter that orchestrates the transformation of pixel values in images to achieve enhanced contrast and improved visual perception. UCEV introduces an adaptive mechanism to adjust pixel values, broadening or contracting their distribution across the color space, thereby intensifying contrast between different features or addressing issues like overly bright regions. This concept hinges on dynamically manipulating pixel distributions while maintaining image integrity, offering adaptability and versatility across various image types and contexts. UCEV’s automated approach eliminates the need for manual parameter adjustment, optimizing image quality by achieving a balance between contrast enhancement and preservation of image details through interaction with entropy maximization, resulting in enhanced visual interpretation and improved image quality.

UCEV introduces an adaptive mechanism, denoted as $\alpha$ influencing each new pixel value $x^{\prime}_{i}$, as articulated through the following Eq. (8).

8 $$x^{\prime}_{i} = x_{i} + \alpha \sum\limits_{j = 1}^{k} {u_{ij} (x_{i} - c_{j} )\forall i = 1....n}$$

where $\alpha$ symbolizes the step size in a line search, leading away from the central cluster points through a fusion of fuzzy directions. A positive value of $\alpha$ widens the image dispersion, resulting in heightened contrast enhancement. Conversely, a negative $\alpha$ value signifies the contraction of pixel dispersion, potentially aiding in the adjustment of excessively bright photographs. Within the context of contrast enhancement, the Eq. (8) serves as an indispensable tool for harmonizing pixel colours. This study is specifically centred on assessing performance under the condition of K = 3. However, it is imperative to acknowledge the need for future investigations to ascertain the optimal clustering parameter. Furthermore, it becomes evident that achieving a brighter photograph with enhanced contrast and harmonious colour distribution is attainable by utilising real number values within the interval of [0, 1] for $u_{ij}$.

A global measurement, entropy *J*^45^ is maximized concerning $\alpha$, which is the final step of contrast enhancement shown by the Eq. (9).

9 $$\underbrace {Max}_{\alpha }J(\alpha ) = - \sum\limits_{x \in \Omega } {[p(x^{\prime}(\alpha )} )\log_{2} p(x^{\prime}(\alpha ))]$$

where $p$ stands for probability density and $x^{\prime}$ a set of image pixels which was obtained from the Eq. (8) for measuring the unpredictability of distribution of pixels. The analysis is conducted through the application of a gridding set $\Omega$, which is employed for the computation of image entropy. A higher entropy value represents an increased degree of randomness within pixel distribution, showcasing substantial variance between the pixels. This results in enhancing image contrast by the optimization process stated in the Eq. (9).

**Figure 2:**
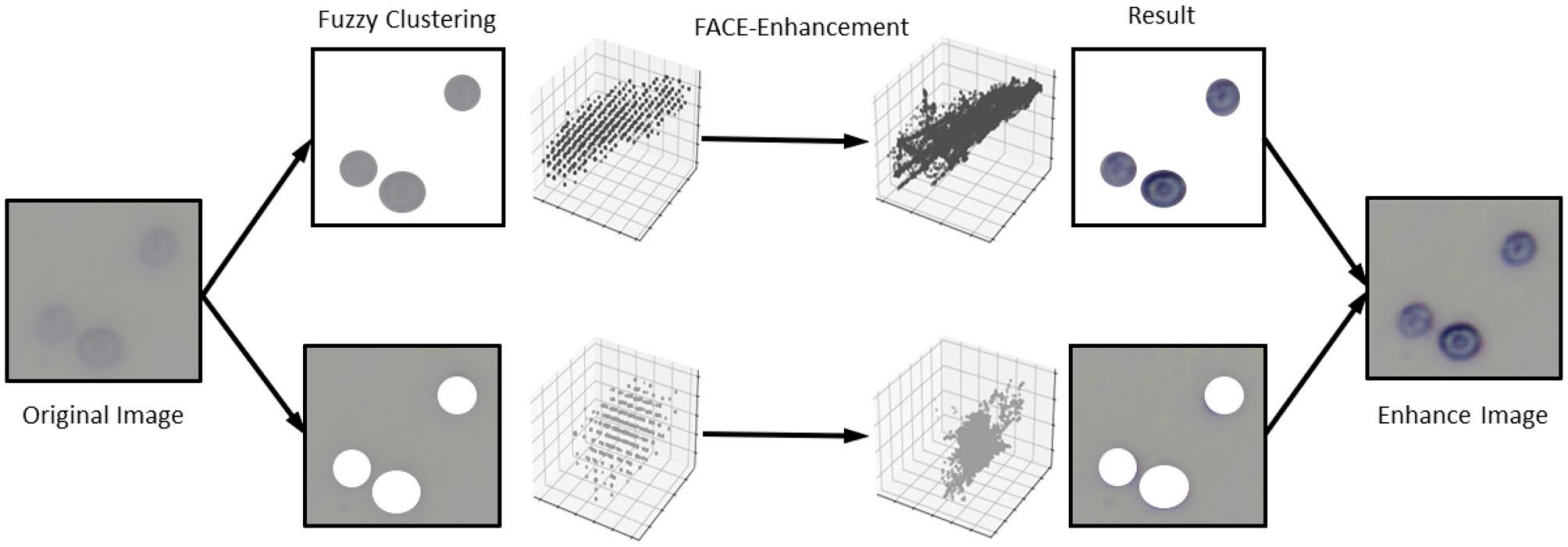
FACE process of yeast cell.

Within the analyzed colour space, the expansion of the pixel distribution is curtailed. In this study, the repositioned pixel points that exceed the boundaries of the colour space are constrained to remain within those limits. As a consequence, the escalation in image entropy ceases once specific pixel points converge upon the boundary edges. A higher value of $\alpha$ will lead to a greater accumulation of pixel points clustering along the bounds, resulting in reduced variation among them and potentially yielding a lower level of entropy. Consequently, the application of the maximization technique yields maximal entropy. An illustration of the FACE process as applied to yeast cells is depicted in Fig. 2.

### Demonstration of the FACE enhancement for Yeast Cells

**Figure 3:**
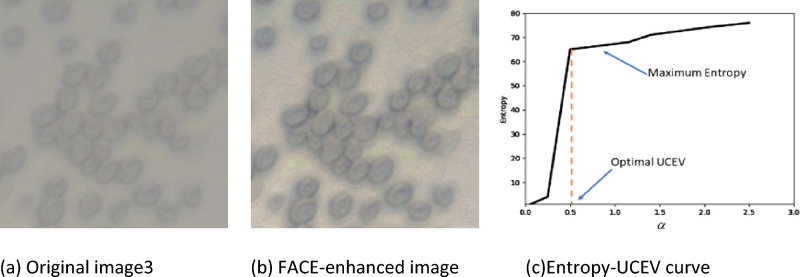
FACE-Entropy maximization process. (**a**) Original image3, (**b**) FACE-enhanced image, (**c**)Entropy-UCEV curve.

An insightful observation has been presented, highlighting the significance of determining the optimal UCEV value ($\alpha$). This value intricately ties in with the accumulation of pixel points along the image boundaries, subsequently influencing the entropy. When UCEV provides a higher value, it corresponds to the maximization of entropy. The entire process of enhancement with the relation of $\alpha$ and entropy are illustrated in Table 1. The first row within the table visually maps out the FACE enhancement procedure, encompassing alterations in the $\alpha$ value. The accompanying graph elegantly portrays the resulting variation in entropy as a function of the $\alpha$ value, providing a comprehensive visual representation of this process. Therefore, by employing the maximization process embedded in FACE, the optimal value of UCEV can be identified, leading to the attainment of maximum entropy. This endeavour significantly enhances image quality, as visually depicted in Fig. 3.

**Table 1:**
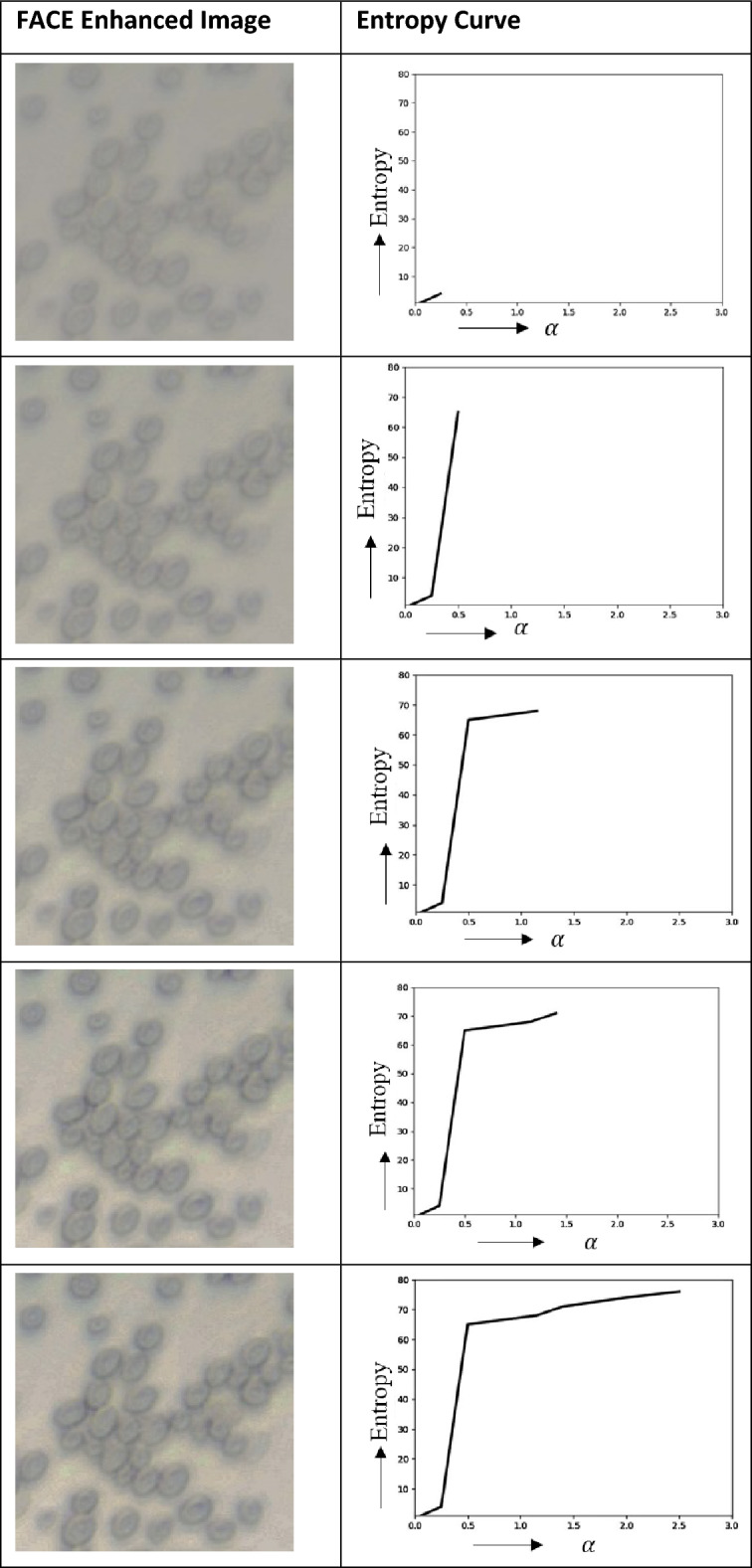
Variation of FACE contrast-enhanced images with entropy curve.

**Figure 4:**
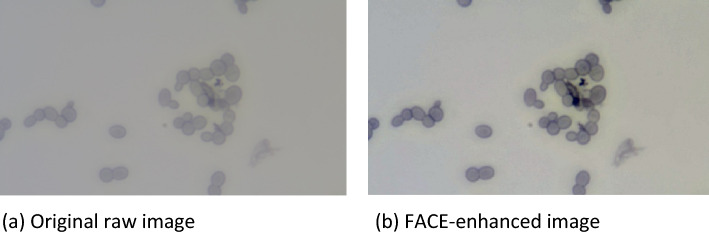
Contrast enhancement of the microscopic image of yeast cell. (**a**) Original raw image, (**b**) FACE-enhanced image.

Figure 4a shows the original raw microscopic image of yeast cells to demonstrate the contrast enhancement. The suggested FACE approach uses fuzzy clustering and fuzzy entropy maximization to determine the optimal conditions for the contrast enhancement of the image. The smooth enhancement is accomplished by the fuzziness in the clustering process, which assures the belongingness of the pixel point’s accuracy. With just one control parameter, the specification of UCEV enables the contrast enhancement process could efficiently produce a global contrast enhancement effect. The entropy maximization technique assures that the pixel distributions are greatly enlarged and do not gather on the color space’s boundaries. An enhanced image is optimized by the proposed FACE method, as shown in Fig. 4b, and it is determined without the need for human feed control parameters.

### Enhancement performance measurement

Two statistical methods are used to measure the qualitative index of contrast enhancement performed by FACE and other techniques. The first method is the Root-Mean-Square Deviation (RMSD), which was performed between the original and enhanced images. The Eq. (10) shows the calculation for the RMSD value.

10 $$RMSD = \sqrt {\frac{1}{n}\sum\limits_{i = 1}^{n} {\mathop {\left\| {x^{\prime}_{i} - x_{i} } \right\|}\nolimits^{2} } }$$

where $x_{i}$ and $x^{\prime}_{i}$ are the $i{\text{th}}$ pixel point of the original image and enhanced image, respectively, in case of a large number of RMSD values shows the large variation in pixel color caused by the method used for enhancement. If the original image has low contrast, it is desired to have an enhanced image with a greater RMSD value. If the case is reversed, then the value of RMSD would be desirably small.

Another method known as Root-Mean-Square Contrast (RMSC) is used for quantifying the contrast enhancement process, where the difference between each pixel point is calculated as shown in the Eq. (11).

11 $$RMSC = \sqrt {\frac{1}{n}\sum\limits_{i = 1}^{n} {\left\| {x^{\prime}_{i} - \sum\limits_{k = 1}^{n} {x^{\prime}_{k} } } \right\|}^{2} }$$

Spatial distribution of contrast does not rely on the measurement of the RMSC, which means that it cannot provide the existence of artifacts. This method provides an overall impression of the image feature and its background. An image with a higher RMSC is desirable, but if its value is too high, it will be over-enhanced.

### Automatic cell detection using YOLOv5

#### Yolov5

YOLOv5 is a single-stage object detector containing three crucial components like another single-stage detector. The components of YOLOv5 are the model backbone, model neck, and model head. Model backbone is the initial stage, whose primary purpose is to extract significant features from an input image. In YOLOv5, the cross-stage partial networks (CSP) are applied as a backbone to extract rich, informative features from an input image. With deeper networks, CSPNet has demonstrated a considerable reduction in processing time.

**Figure 5:**
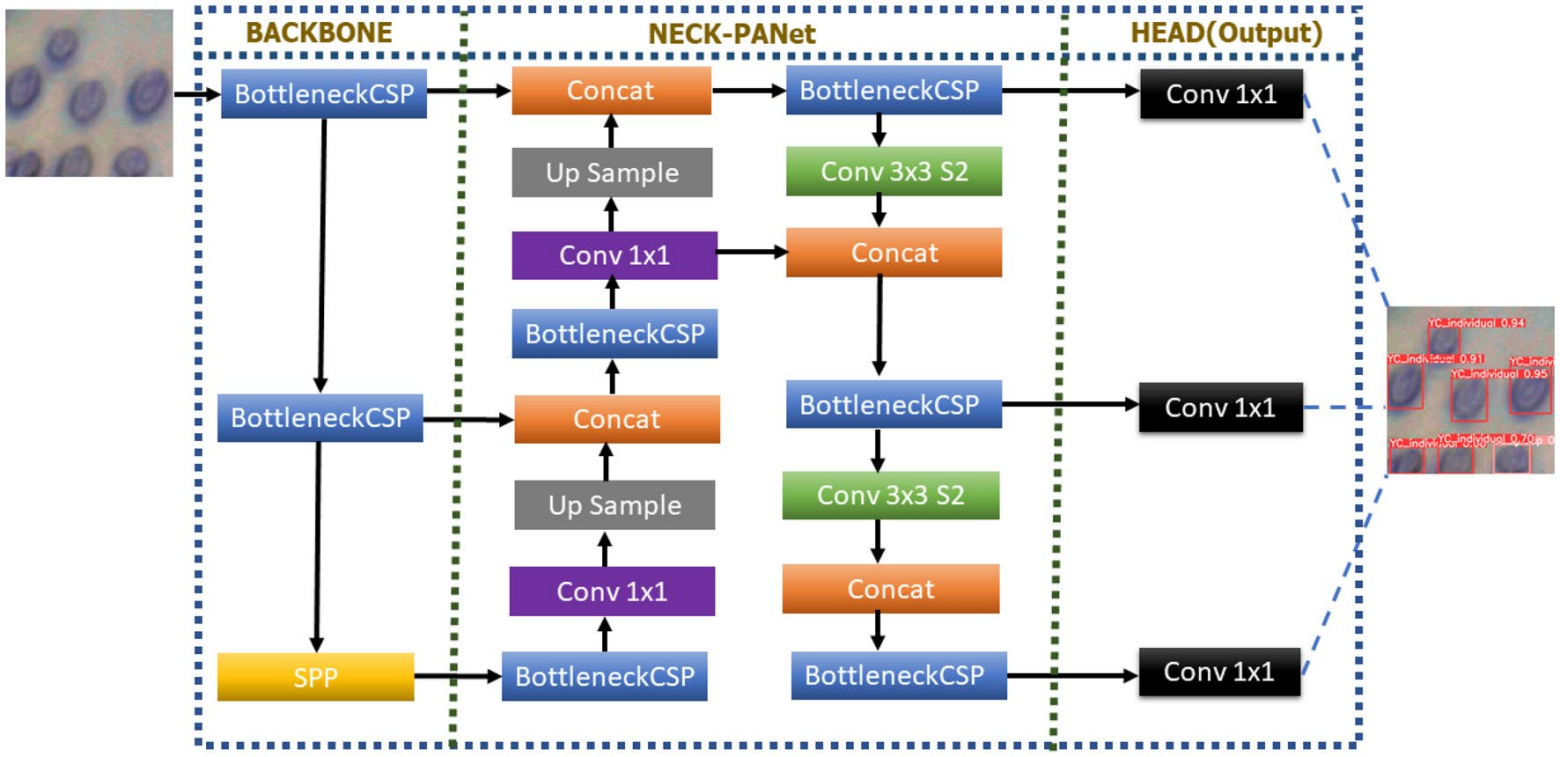
Architecture of YOLOv5.

YOLOv5 has a second component known as the model neck, which generates feature pyramids. Feature pyramids assist models in developing decent object scaling generalizations. Model neck helps to recognize the same object in various sizes and scales. Models that employ feature pyramids perform well on unseen data. Different feature pyramid methods are available such as BiFPN, PANet, FPN, etc. PANet is a feature pyramid technique that is used in YOLOv5. The model head performs the final detection part. Anchor boxes are applied to features through which final output vectors are generated along with class probabilities, objectness, and bounding boxes. The model head is the same as in previous YOLO versions. The final model architecture for YOLOv5 is shown in Fig. 5.

YOLOv5 was used to recognize the yeast cells in the investigated images. YOLOv5 is an object detection method based on CNN structure. The confidence level of each recognized image pattern based on CNN is evaluated, and the optimal bounding box of a recognized image pattern is then determined. The yeast cell image patterns learning database in this paper is built on the tool Labellmg and Roboflow.

### Automatic cell detection using FACE-YOLO model

**Figure 6:**
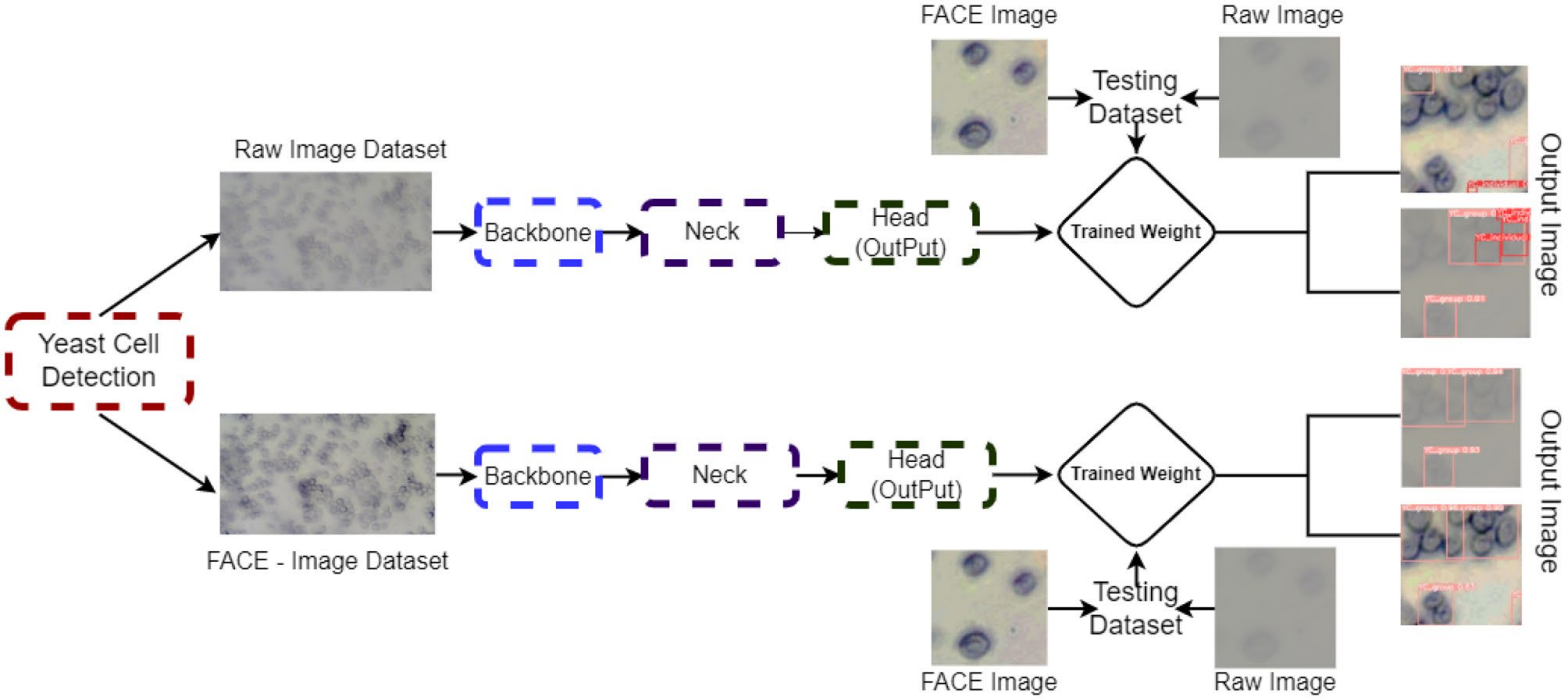
Yeast cell detection and classification using FACE-YOLO model.

To comprehensively investigate the impact of contrast enhancement in automatic yeast cell detection, our study meticulously created two sets of image databases. The first encompassed the original raw yeast cell images, while the second was composed of meticulously enhanced images utilizing the FACE approach. This dual-pronged approach enabled us to intricately probe the effect of contrast enhancement on the efficacy of the yeast cell detection methodology, seamlessly integrated with the cutting-edge YOLOv5 framework. The process of automatic cell detection using this FACE-YOLO model was shown by a schematic diagram shown in Fig. 6.

The yeast cell detection process commences with the meticulous labelling and organization of the dataset. To facilitate this, two distinct datasets are meticulously curated: the initial dataset comprising raw cell images and an additional dataset comprising images of yeast cells subjected to enhancement via the FACE methodology. Subsequently, the labelled training images are allocated to the backbone, neck, and head of the architecture, culminating in the generation of the desired output. The weights acquired through the training process are then applied to the testing phase, yielding conclusive results. Detection is executed through systematic manipulation of the input images and datasets. Initially, the raw training dataset is employed for cell detection, involving both raw images and FACE-enhanced images. Following this, the training dataset enriched with FACE-enhanced images is deployed for cell detection, encompassing both the original raw images and their enhanced counterparts. The computational parameter used for the FACE-YOLO model is detailed in Table 2.

**Table 2:** Parameter for FACE-Yolo Model.

	Input dimensions	Network architecture	Optimization algorithm	Learning rate	batch size	Epochs
FACE-Yolo model	640 × 640	CNN-based	SGD	0.001	46	300

A comparative analysis presented in Table 3 scrutinises the efficiency of training and testing interchange utilizing original raw images and FACE-enhanced images for yeast cell detection. The results illuminate that training with FACE-enhanced images consistently yields superior detection performance automatic cell detection with YOLOv5 for both raw and enhanced image datasets.

**Table 3:**
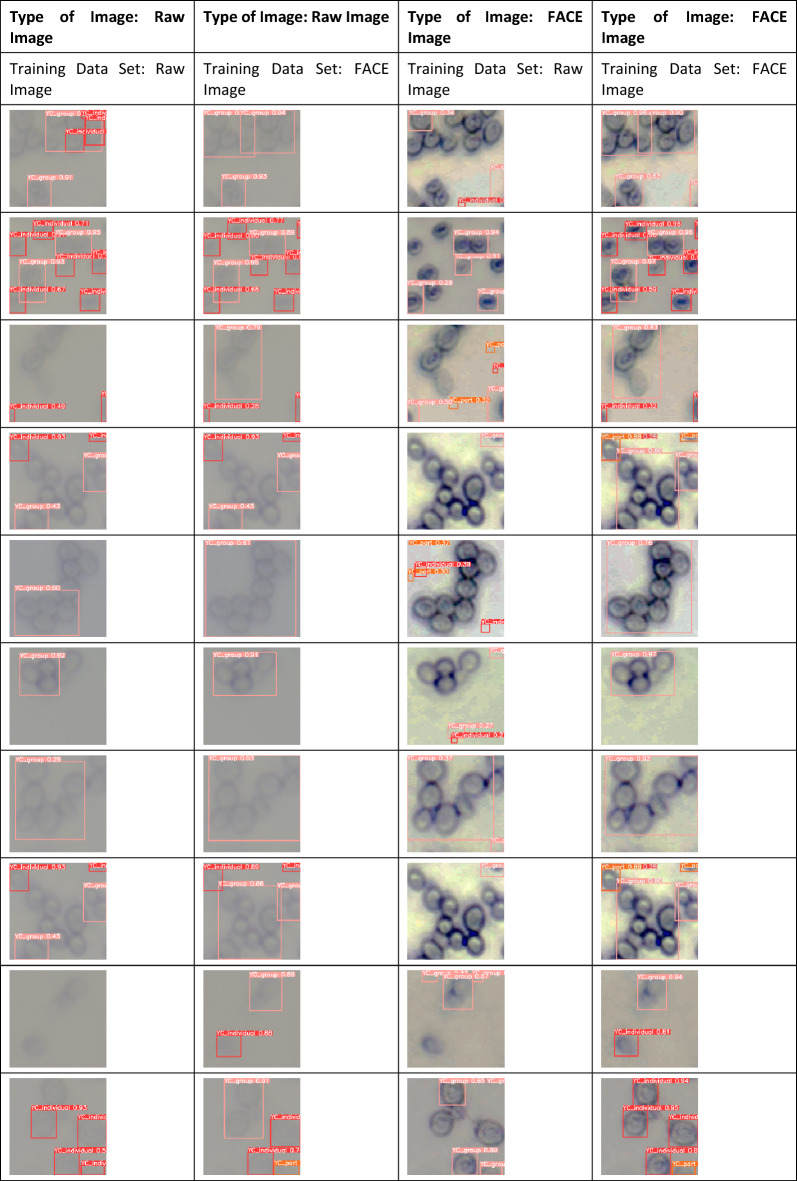
YOLOv5 detections for raw image and FACE enhanced image by using a different training set.

**Figure 7:**
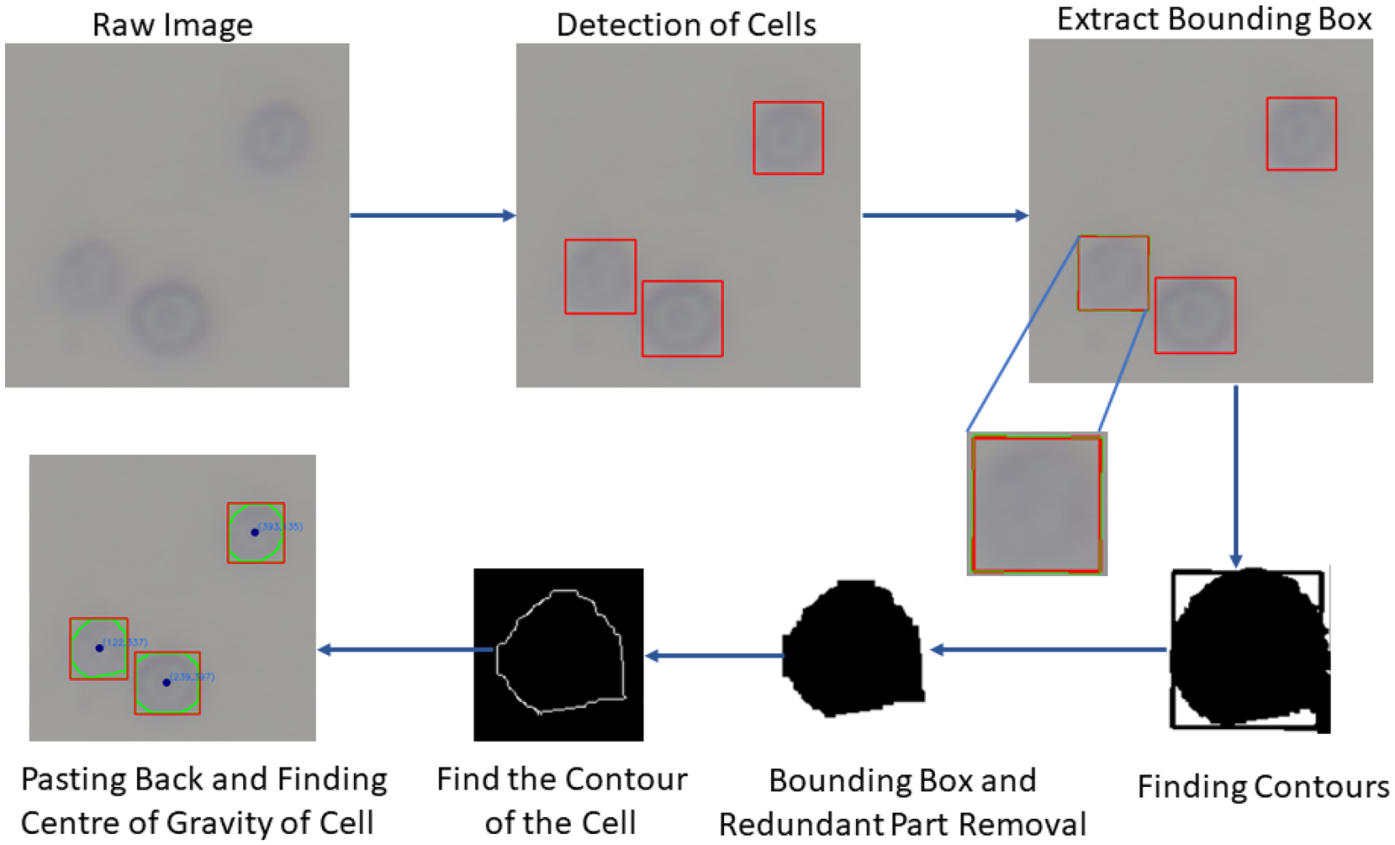
Process of determination of centre and boundary of cells.

For the task of finding the real-time position of the cell, the primary objective is to get the contour and pinpoint the precise coordinates of the yeast cell. This dynamic real-time process for detecting yeast cell contour and centre of the cell is illustrated in Fig. 7. The process unfolds with the utilization of YOLOv5 to train the FACE dataset, followed by employing the trained FACE model to detect yeast cells in the raw image. Subsequently, the application of OpenCV comes into play to identify the contour and location of each yeast cell. The procedure commences by extracting the bounding box from the YOLOv5 image’s outcome to verify the yeast cell’s position. Subsequent steps involve transforming the image into binary form, eliminating the bounding box and redundant portions, and employing the OpenCV function ‘cv2’ to locate the cell’s contour and centre. Finally, the identified contour and centre are overlaid onto the original raw image.

## Results analysis

The microscopic images of yeast cells are taken from the laboratory, creating a database of 1000 raw images. This paper focuses on the performance of fuzzy clustering at $k = 2$. Comparative analysis is done using six original images from the database using three different techniques: FACE, SSR, and HE. The comparison results of contrast enhancement of the original image with other methods as shown in Table 4.

**Table 4:**
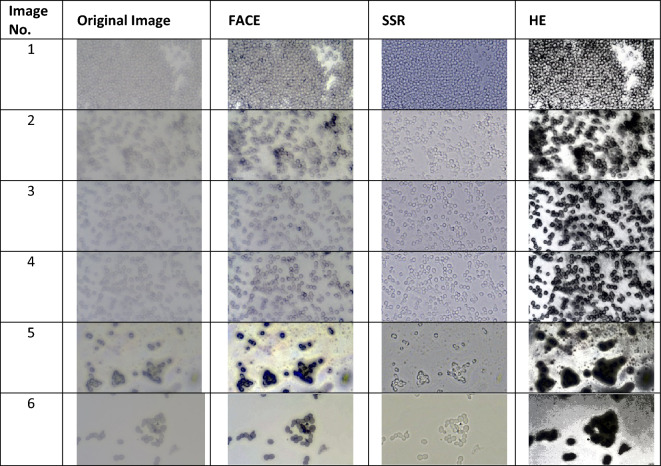
Comparison of contrast enhancement using different methods.

The qualitative index was measured using RMSD and RMSC values for original microscopic and enhanced images, shown in Table 4. RMSC values of original and enhanced images were calculated, shown in Table 5. RMSC values have increased after the image enhancement using different techniques. The RMSC of all the enhanced images is higher than the RMSC of the Original image. But when compared, HE has a very high RMSC value which provides over-contrast enhancement. While we can observe that features are apparent using FACE. Using an SSR image has generated a greater amount of color change than the original image. Many artifacts were ground in the background when SSR and HE were used. Hence fuzzy clustering in FACE keeps the image free of artifacts and provides good features that keep the image’s originality.

**Table 5:** RMSC values of the original images and enhanced images.

Enhancement methods	Image 1	Image 2	Image 3	Image 4	Image 5	Image 6
Original	7.03	7.43	6.02	5.83	14.45	7.43
FACE	17.06	16.71	12.39	12.031	37.01	17.78
SSR	25.64	14.81	15.77	17.58	15.89	9.28
HE	72.4	74.93	74.57	74.43	42.03	75.65

While comparing the RMSD values, Table 6 shows that FACE provides a lower RMSD value than HE and SSR.FACE does not overchange the image’s contrast to the original image, while the RMSD value is small compared to the other two methods, which shows that the other techniques have changed the colour tones and features lacking issues compared with the original image. Hence FACE delivers the proper amount of contrast enhancement for the microscopic images.

**Table 6:** RMSD values of enhanced images.

Enhancement methods	Image 1	Image 2	Image 3	Image 4	Image 5	Image 6
FACE	10.95	9.59	6.73	6.43	26.5	16.015
SSR	32.96	27.07	14.86	22.72	15.44	15.623
HE	18.45	22.86	26.05	25.69	67.35	71.151

**Figure 8:**
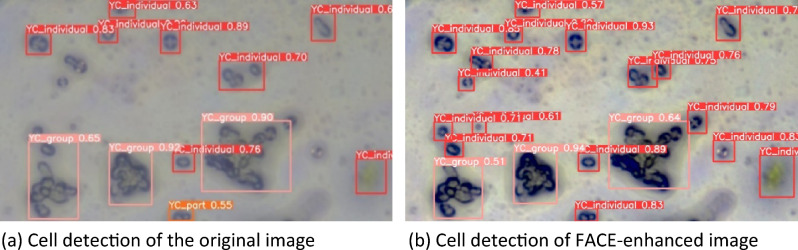
Yeast cell detection using YOLOv5 of microscopic images. (**a**) Cell detection of the original image, (**b**) Cell detection of FACE-enhanced image.

Next, YOLOv5 was used to recognize the yeast cells from the original microscopic images. In this study, the two models using a training dataset of the yeast cell image patterns are generated, and the effect of image enhancement on object detection by YOLOv5 is analyzed. The first model using the dataset was original raw yeast cell images, and the second model the dataset of enhanced images by FACE. Each model uses a different dataset to build a YOLO object detection system. When the performance compared for the detection of yeast cells between the original raw image model and the FACE model, it is observed that when the original raw image model is considered for YOLO detection, many cells are not identified due to being small in nature or blurred image, as shown in Fig. 8a. On the other hand, the FACE model could effectively increase the visual quality of the cells in the image, as shown in Fig. 8b, and improve object detection accuracy. As observed from the two models of cell detection using YOLOv5 for comparative analysis, the FACE model provides good results with higher accuracy of yeast cell detection using YOLOv5, and it is also capable of detecting the yeast cells with original raw input image having low contrast features with high accuracy.

The performance of the FACE training model was analyzed using tenfold cross-validation accuracy and confidence score. As we can see, in the first fold and the second fold of the accuracy, the detection of the FACE training model had an accuracy above 90%, as compared with the detection of the raw image training model accuracy the FACE training model is more suitable for the blurry image or boundary of the cells are not clear. The confidence scores of the FACE training model are much higher than the raw training model, although the third and the fifth fold are lower, and these two confidence scores do not differ much. So the confidence scores of the FACE model are nearly the highest among the two models. The tenfold cross-validation accuracy and confidence scores are shown in Table 7.

**Table 7:** Accuracy and Confidence scores.

Accuracy			Confidence	
Folds	RAW	FACE	RAW	FACE
1	0.81	0.90	0.211	**0.691**
2	0.88	0.91	0.361	0.607
3	0.90	0.94	**0.629**	0.609
4	0.94	0.92	0.514	**0.726**
5	0.98	0.96	**0.764**	0.762
6	0.94	0.95	0.502	**0.884**
7	0.99	0.98	0.716	**0.854**
8	0.97	0.97	0.392	**0.776**
9	0.97	0.95	0.591	**0.86**
10	0.91	0.94	0.431	**0.681**
Average	0.932	**0.942**		

Significance values are in Bold.

**Figure 9:**
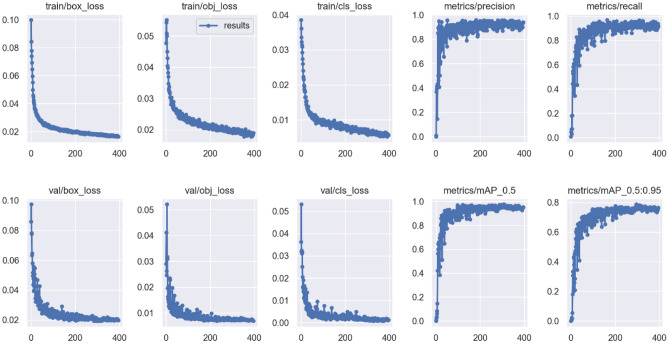
Performance metrics for the FACE training model.

The image shown in Fig. 9 is the performance metrics of the FACE training model, resulting in the loss value of train and validation convergence gradually and then reaching a stable level, and the precision, recall, and mAP value rise to high accuracy and reach almost 100%. The training leads to a satisfactory result that shows our model is trained well.

**Figure 10:**
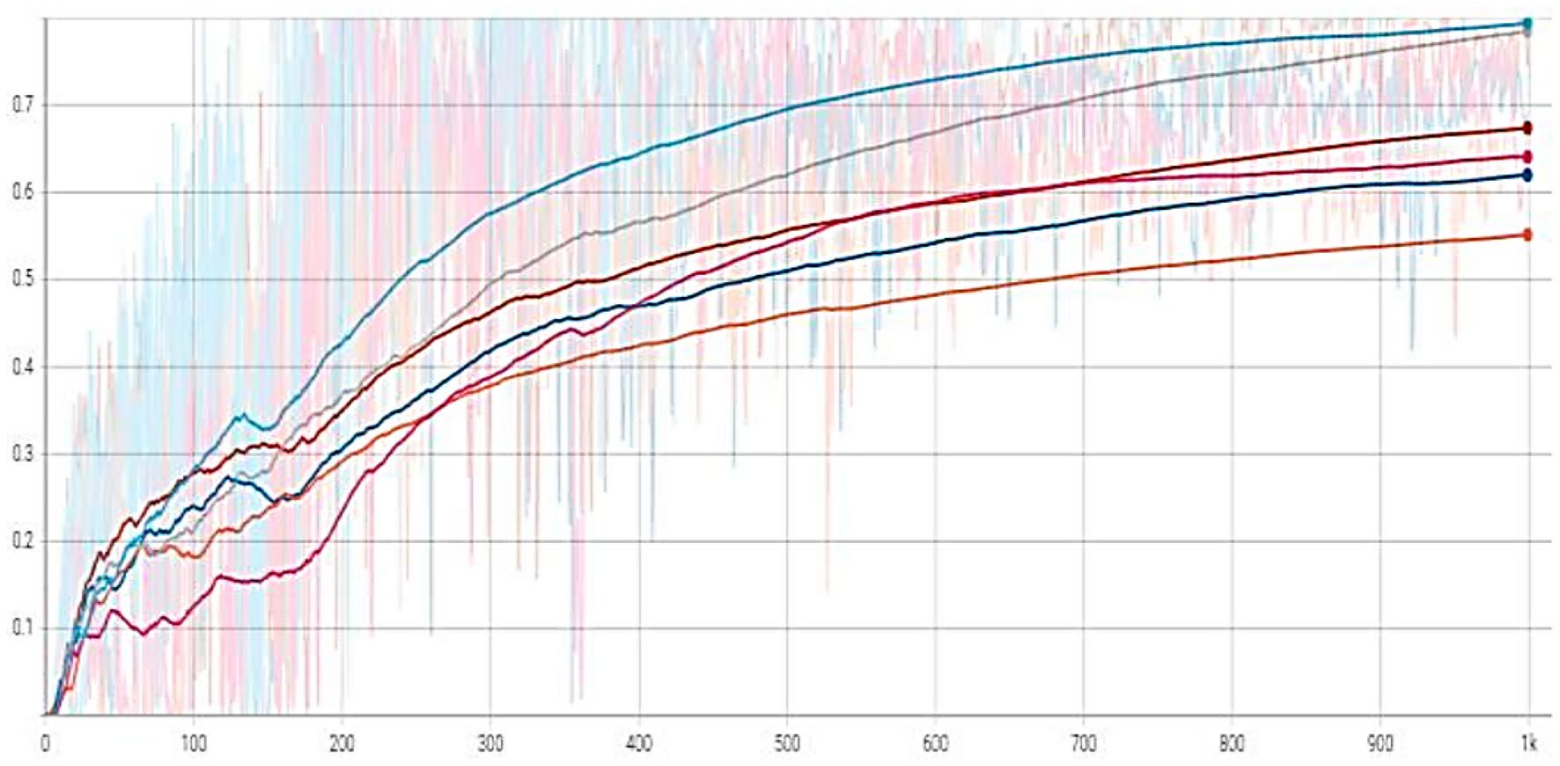
Detection accuracy comparison of YOLOv5.

The recognition accuracy is demonstrated using different raw, enhanced, and synthetic training datasets. The synthetic dataset is a computer-generated yeast cell image from Blender3D^46^ representing all the object classes. It is noted that the boundaries of the yeast cells in the enhanced images are more precise and accessible to recognize than those in the original images. At first, the model is trained with 100% raw images to set a reference result. Then the next model is trained repeatedly, and for each new session, 10% of the dataset is switched out to enhanced images until the training dataset is 100% enhanced images. The same was done with the synthetic dataset. Then the following models are trained with different enhanced: synthetic dataset ratios. For determining the quality of the results, we used metrics such as mAP_0.5. The recognition accuracy demonstrated by the dark blue curve, which is found to be low, was based on the original microscopic images of yeast cells. When the accuracy is performed using 40% synthetic, and 60% enhanced images, it provides good detection accuracy, shown by the curve in grey color. The other curves are based on different research settings and can be seen in Fig. 10.

**Figure 11:**
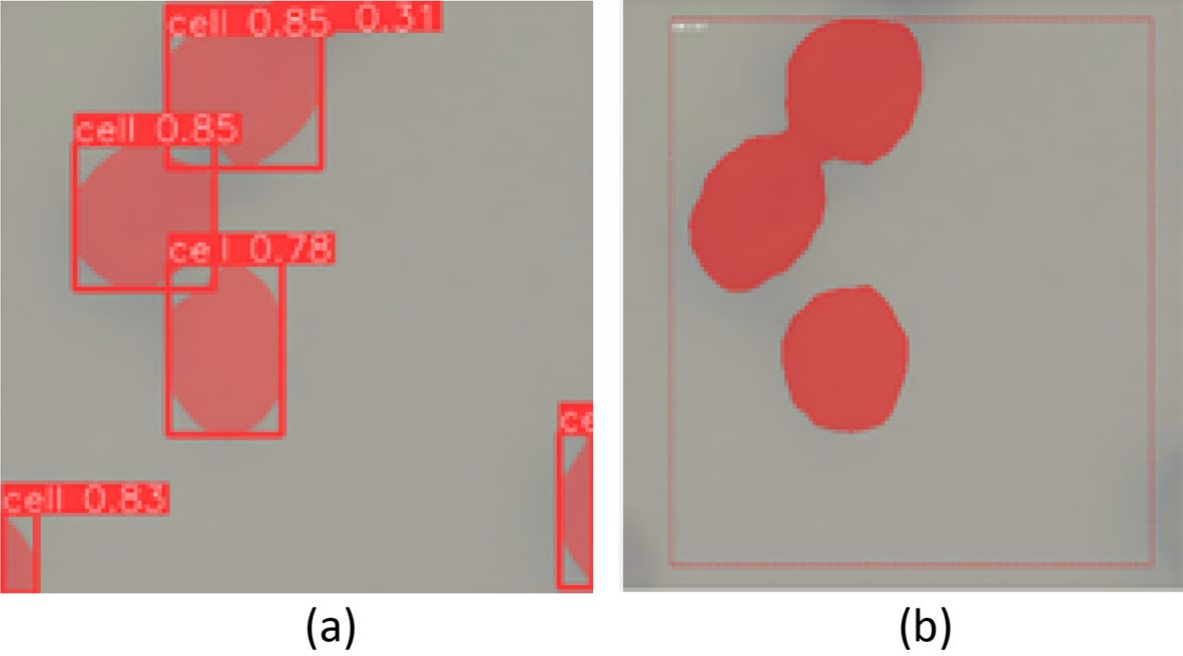
Automatic yeast cell detection (**a**) FACE-YOLO model (**b**) Mask R-CNN

In the pursuit of enhancing the efficiency of the proposed model, a comparative study was undertaken involving two state-of-the-art deep learning models: FACE-YOLO and Mask R-CNN, for the task of automated yeast cell detection. The tenfold accuracy results of both models are presented in Table 8, revealing that the FACE-YOLO model outperforms Mask R-CNN in terms of accuracy. The comparitive outcomes of automatic cell detection by FACE-YOLO and Mask R-CNN are visually depicted in the accompanying Fig. 11.

**Table 8:** 10 Fold Accuracy of FACE-YOLO and Mask R-CNN.

Folds	FACE-YOLO	Mask R-CNN
1	0.84	0.89
2	0.92	0.93
3	0.90	0.92
4	0.94	0.90
5	0.91	0.89
6	0.95	0.91
7	0.98	0.81
8	0.97	0.92
9	0.95	0.91
10	0.94	0.89
Average	0.942	0.897

## Conclusion

This work proposes Fuzzy Automatic Contrast Enhancement (FACE) as an image quality enhancement technique to enhance the microscopic images of yeast cells. FACE employs fuzzy clustering to prevent visible pixel separation, while a universal contrast enhancement variable (UCEV) optimizes entropy for enhanced image quality. In this work, the image enhancement of the microscopic image of yeast cells was done automatically without any feed of human-defined parameters. Finally, FACE created a less congested image pixel distribution with a significantly larger picture contrast while effectively enhancing the yeast cell image’s contrast level without producing local flaws such as a change in colour, visual artifacts, and over-strengthening. When the qualitative index was performed, and compared to other methods, FACE performed better enhancement results. The integration of YOLOv5 for automatic yeast cell detection further elevates the study's contributions. Notably, the FACE-enhanced model within YOLOv5 improves yeast cell detection accuracy. Impressively, the FACE-YOLO model outperforms Mask R-CNN with a remarkable 94.2% accuracy. This study underscores the promising synergy between FACE-enhanced imagery and advanced detection techniques, offering a pathway for heightened precision and effectiveness in cell analysis across a spectrum of biomedical applications. Additionally, the localization of cell boundaries and centres through OpenCV enhances cell positioning accuracy. As a future direction, integrating the automatic FACE-YOLO cell detection with a microgripper robot holds the potential for facilitating cell manipulation and handling.

## Data Availability

The datasets used during the current study are available from the corresponding author upon request.

## References

[1] Wang H (2018). Biological image analysis using deep learning-based methods: Literature review. Digit. Med..

[2] Eliceiri KW (2012). Biological imaging software tools. Nat. Methods.

[3] Liu J, Fan Z, Zhao W, Zhou X (2021). Machine intelligence in single-cell data analysis: advances and new challenges. Front. Genet..

[4] Simon I (2001). Serial regulation of transcriptional regulators in the yeast cell cycle. Cell.

[5] Iyer VR (2001). Genomic binding sites of the yeast cell-cycle transcription factors SBF and MBF. Nature.

[6] Rustici G (2004). Periodic gene expression program of the fission yeast cell cycle. Nat. Genet..

[7] Kulwa F (2019). A state-of-the-art survey for microorganism image segmentation methods and future potential. IEEE Access.

[8] Bredies K, Wolinski H (2011). An active-contour based algorithm for the automated segmentation of dense yeast populations on transmission microscopy images. Comput. Visual Sci..

[9] Casacio CA (2021). Quantum-enhanced nonlinear microscopy. Nature.

[10] Rani P, Kotwal S, Manhas J, Sharma V, Sharma S (2022). Machine learning and deep learning based computational approaches in automatic microorganisms image recognition: Methodologies, challenges, and developments. Arch. Comput. Methods Eng..

[11] Gonzalez, R. C. *Digital image processing*. (Pearson education INDIA, 2009).

[12] Demirel H, Ozcinar C, Anbarjafari G (2009). Satellite image contrast enhancement using discrete wavelet transform and singular value decomposition. IEEE Geosci. Remote Sens. Lett..

[13] Lin, P. T. & Lin, B. R. in *2016 12th IEEE/ASME International Conference on Mechatronic and Embedded Systems and Applications (MESA).* 1–10 (IEEE).

[14] Redmon, J., Divvala, S., Girshick, R. & Farhadi, A. in *2016 IEEE Conference on Computer Vision and Pattern Recognition (CVPR).* 779–788.

[15] Chaker A, Mlika A, Laribi M, Romdhane L, Zeghloul S (2013). Clearance and manufacturing error's effects on the accuracy of the 3-RCC Spherical Parallel Manipulator. Eur. J. Mech. A/Solids.

[16] Dietler N (2020). A convolutional neural network segments yeast microscopy images with high accuracy. Nat. Commun..

[17] Pizer SM (1987). Adaptive histogram equalization and its variations. Comput. Gr. Image Process..

[18] Abdullah-Al-Wadud M, Kabir MH, Dewan MAA, Chae O (2007). A dynamic histogram equalization for image contrast enhancement. IEEE T Consum. Electr..

[19] Pisano ED (1998). Contrast limited adaptive histogram equalization image processing to improve the detection of simulated spiculations in dense mammograms. J. Digit. Imaging.

[20] Land EH, McCann JJ (1971). Lightness and retinex theory. J. Opt. Soc. Am..

[21] Jobson DJ, Rahman Z-U, Woodell GA (1997). Properties and performance of a center/surround retinex. IEEE Trans. Image Process..

[22] Fu, X., Zeng, D., Huang, Y., Zhang, X.-P. & Ding, X. in *2016 IEEE Conference on Computer Vision and Pattern Recognition (CVPR).* 2782–2790.

[23] Guo X, Li Y, Ling H (2016). LIME: Low-light image enhancement via illumination map estimation. IEEE Trans. Image Process..

[24] Rahman, Z.-u., Jobson, D. J. & Woodell, G. A. in *Proceedings of 3rd IEEE International Conference on Image Processing.* 1003–1006 (IEEE).

[25] Bezdek JC, Ehrlich R, Full W (1984). FCM: The fuzzy c-means clustering algorithm. Comput. Geosci..

[26] Dileep, M. & Murthy, A. S. in *2011 International Conference on Emerging Trends in Electrical and Computer Technology.* 708–712 (IEEE).

[27] Ionescu, C., Fosalau, C. & Petrisor, D. in *2014 International Conference and Exposition on Electrical and Power Engineering (EPE).* 100–104 (IEEE).

[28] Alanazi A (2022). Using machine learning for healthcare challenges and opportunities. Inform. Med. Unlocked.

[29] Kaul, D., Raju, H. & Tripathy, B. Deep learning in healthcare. *Deep Learning in Data Analytics: Recent Techniques, Practices and Applications*, 97–115 (2022).

[30] Ghafari, M. *et al.* in *2022 International Conference on Artificial Intelligence in Information and Communication (ICAIIC).* 204–209 (IEEE).

[31] Maddalena L, Antonelli L, Albu A, Hada A, Guarracino MR (2022). Artificial intelligence for cell segmentation, event detection, and tracking for label-free microscopy imaging. Algorithms.

[32] Li C, Wang K, Xu N (2019). A survey for the applications of content-based microscopic image analysis in microorganism classification domains. Artif. Intell. Rev..

[33] Rea D, Perrino G, di Bernardo D, Marcellino L, Romano D (2019). A GPU algorithm for tracking yeast cells in phase-contrast microscopy images. Int. J. High Perform. Comput. Appl..

[34] Zeng Z, Xie W, Zhang Y, Lu Y (2019). RIC-Unet: An improved neural network based on Unet for nuclei segmentation in histology images. IEEE Access.

[35] Moen E (2019). Deep learning for cellular image analysis. Nat. Methods.

[36] Hilsenbeck O (2017). fastER: a user-friendly tool for ultrafast and robust cell segmentation in large-scale microscopy. Bioinformatics.

[37] Wang B, Cao G, Zhou L, Zhang Y, Shang Y (2020). Task differentiation: Constructing robust branches for precise object detection. Comput. Vis. Image Underst..

[38] Yang, S. *et al.* in *2017 International Conference on Security, Pattern Analysis, and Cybernetics (SPAC).* 345–350 (IEEE).

[39] Fujita, S. & Han, X. -H. in *Proceedings of the Asian Conference on Computer Vision.*

[40] Bresilla K (2019). Single-shot convolution neural networks for real-time fruit detection within the tree. Front. Plant Sci..

[41] Jain AK (2010). Data clustering: 50 years beyond K-means. Pattern Recognit. Lett..

[42] Wagstaff, K., Cardie, C., Rogers, S. & Schrödl, S. in *Proceedings of the Eighteenth International Conference on Machine Learning.* 577–584 (Morgan Kaufmann Publishers Inc.).

[43] Cannon RL, Dave JV, Bezdek JC (1986). Efficient implementation of the fuzzy c-means clustering algorithms. IEEE Trans. Pattern Anal. Mach. Intell..

[44] Otsu N (1979). A threshold selection method from gray-level histograms. IEEE T Syst. Man. Cyb.

[45] Narayan R, Nityananda R (1986). Maximum entropy image restoration in astronomy. Annu. Rev. Astronomy Astrophys..

[46] Community B (2018). Blender–A 3D Modelling and Rendering Package.

